# Mismatch of corneal specialists’ expectations and keratoconus knowledge in general ophthalmologists - a prospective observational study in Switzerland

**DOI:** 10.1186/s12909-021-02738-0

**Published:** 2021-05-25

**Authors:** Philipp B. Baenninger, Lucas M. Bachmann, Katja C. Iselin, Oliver A. Pfaeffli, Claude Kaufmann, Michael A. Thiel, Gerd Gigerenzer

**Affiliations:** 1 Faculty of Medicine, University of Zurich, 8091 Zurich, Switzerland; 2 Department of Ophthalmology, Cantonal Hospital of Lucerne, 6000 Lucerne-16, Switzerland; 3 Medignition Inc. Research Consultants Zurich, Verena Conzett-Strasse 9, PO 9628, 8036 Zurich, Switzerland; 4 Max Planck Institute for Human Development, Lentzeallee 94, 14195 Berlin, Germany

**Keywords:** Medical education, minimal knowledge, keratoconus, general ophthalmologist, mismatch, interview survey

## Abstract

**Background:**

To assess whether Swiss general ophthalmologists have the minimal keratoconus knowledge that corneal specialists would expect them to have.

**Methods:**

Corneal specialists defined “minimal keratoconus knowledge” (MKK) with respect to definition, risk factors, symptoms and possible treatment options of keratoconus. A telephone interview survey was conducted among one hundred ophthalmologists (mean age 51.9 years (SD 9.5), 60 % male) from the German-speaking part of Switzerland. For each participant, years of work experience, number of keratoconus patients seen per year and access to a topography device were obtained. We calculated the proportion of MKK and examined in multivariate analyses whether ophthalmologists with access to topography and with greater work experience performed better than other groups.

**Results:**

No single ophthalmologist had MKK. The mean MKK was 52.0 %, and the range was 28.6–81.0 %. Per 10 years of working in private practice, the MKK decreased by 8.1 % points (95 % CI: -14.2, -2.00; p = 0.01). Only 24 % of participants correctly recalled the definition of keratoconus, 9 % all risk factors, 5 % all symptoms and 20 % all treatment modalities. The MKK values were not associated with the number of keratoconus patients seen per year and the availability of topography to diagnose keratoconus.

**Conclusions:**

There is a substantial mismatch between corneal specialist’ expectations and general ophthalmologists’ knowledge about keratoconus. The low recall of symptoms and risk factors may explain why ophthalmologists diagnose relatively few cases of keratoconus, resulting in inefficient care delivery and delayed intervention.

## Background

Keratoconus is a progressive corneal disease in which the cornea, normally round, thins and begins to bulge out in a cone shape. This cone shape deflects light as it enters the eye on its way to the light-sensitive retina, causing distorted vision. Treatment focuses on stopping progression with collagen crosslinking and on visual rehabilitation with glasses, rigid contact lenses or corneal transplants [1]. Keratoconus can be diagnosed clinically by slit-lamp findings. However, clinical manifestations are mainly seen in moderate to severe stages of the disease. Conversely, in the earliest stages of keratoconus, there may be no obvious changes, resulting in the majority of these patients going undiagnosed [2]. In the last two decades, however, there has been a major advancement in knowledge of how to diagnose the disease. Based on Scheimpflug imaging, corneal topography systems produce a 3-D image of the anterior eye segment and provide details of the anterior and posterior corneal surfaces as well as pachymetry [3], allowing ophthalmologists to detect keratoconus at a much earlier stage than was previously possible [4]. The prevalence of keratoconus is generally reported as 1:375 [5] to 1:2000 [6] but varies worldwide: from 0.3 to 100,000 in Russia [7] to 54.5 per 100,000 in the United States [6] to 86 per 100,000 in Denmark[8]. Variability may be due to differences in study methodology and design, environmental or genetic factors, or diagnostic criteria and instruments [9]. In addition, current literature reports a much higher prevalence of up to 1:20 [10], which raises the question of whether there is a high rate of undiagnosed keratoconus patients who do not have access to the necessary care. There are no published data on the prevalence of keratoconus in Switzerland nor on the rate of undiagnosed patients, due to the lack of a national screening programme or a national keratoconus registry. In the Swiss healthcare system, patients can seek advice on vision problems from opticians, general practitioners or general ophthalmologists. Therefore, no general standardised referral practice is in place, and keratoconus patients are typically under the care of their primary board-certified general ophthalmologist and see a corneal specialist at a tertiary referral hospital for topographic diagnosis, assessment of keratoconus progression and advice on treatment options.

Modern keratoconus management requires that patients and their ophthalmologists engage in a shared decision-making process. Shared decision making helps to improve the match between treatment options and patients’ personal values and preferences [11]. However, active participation requires patients to have at least a minimal level of understanding of the disease [12]. In a previous study, corneal specialists defined “minimal keratoconus knowledge” (MKK) regarding definition, risk factors, symptoms and possible treatment options of keratoconus and found a dramatic lack of knowledge in keratoconus patients [13]. Given the evidence for a high rate of undiagnosed keratoconus patients in the general population, we wanted to know if lack of knowledge and awareness of keratoconus in general ophthalmologists in Switzerland could be a contributing factor. By conducting the same survey on MKK with a group of general ophthalmologists, we aimed to find out specifically whether there is a potential mismatch between specialists’ expectations and general ophthalmologists’ knowledge about keratoconus.

## Materials and methods

### Study Design

We conducted a single-center, prospective telephone interview survey at a tertiary referral center in Lucerne, Switzerland with general ophthalmologists in private practice in the German-speaking part of Switzerland.

Inclusion criteria were board-certified ophthalmologists working in a general ophthalmology practice in the German-speaking part of Switzerland who had sufficient German-language skills. The exclusion criterion was inability to follow the German questionnaire due to language comprehension problems. This was not assessed systematically, but interviewers were allowed to exclude such participants at their own judgement.

 We informed eligible ophthalmologists by postal letter about the survey on keratoconus and contacted them 1 week later by telephone to assess their interest in participating. If they verbally consented, we agreed on a specific date to conduct a telephone interview based on the open-ended questionnaire with no time restrictions. We did not record the interview. We enrolled ophthalmologists willing to participate in the study in a prospective and consecutive manner. They were offered no incentives for participation.

### Questionnaire development

In a previous study [13] assessing MKK in patients, a questionnaire was developed on the basis of a literature review and discussions held by a focus group of four corneal specialists and two contact lens-fitting optometrists. They defined the minimal knowledge an average keratoconus patient should have in relation to definition, risks and triggers, symptoms and treatment options of the disease. They were instructed to restrict these to the most common set of characteristics that should be known by every keratoconus patient, excluding uncommon factors or unusual presentations of symptoms. In telephone interviews among ophthalmologists, we employed the same questionnaire than the one used to assess MKK in patients [13]. Although ophthalmologists arguably know more about the condition than patients, we refrained from expanding the questionnaire in order to ensure uniform assessment of patients and physicians. Table 1 defines what we considered minimal knowledge.

**Table 1:** Survey interview questionnaire

Question 1	What is keratoconus?
Correct answers:	- Thinning of the cornea (56 %) - Irregular astigmatism (68 %) - Protrusion of the cornea (64 %)
**Question 2**	**Are there risk factors for the occurrence of keratoconus?**
Correct answers:	- Positive family history (79 %) - Allergies (48 %) - Younger age (29 %)
**Question 3**	**What are triggers for the onset of keratoconus?**
Correct answers:	- Eye rubbing (69 %) - Puberty (16 %) - Pregnancy (4 %)
**Question 4**	**What are symptoms of keratoconus?**
Correct answers:	- Deterioration of vision (short-sightedness, astigmatism) (97 %) - Double vision (shadow vision) (31 %) - Light sensitivity (13 %)
**Question 5**	**What are consequences of untreated keratoconus?**
Correct answer:	- Visual deterioration (86 %) - Inability to wear glasses (65 %) - Inability to wear contact lenses (38 %) - Restriction of current occupation (8 %) - Need for a corneal transplant (36 %)
**Question 6**	**What are treatment options for keratoconus?**
Correct answers:	- Glasses (33 %) - Rigid contact lenses (81 %) - Corneal cross-linking (100 %) - Corneal transplant (72 %)

The MKK is computed in the following way. There are six questions for which the minimal correct answers vary between three and five. The total number of correct answers is 21. The MKK of a person is the total number of correct replies times 100 divided by 21. Please note that MKK measures only the minimal knowledge. Additional knowledge that may be important is not measured. Percentage (%) of correct participants’ (n = 100) answers are displayed in parenthesis

 Besides developing the knowledge questions and obtaining demographic participant information, we planned to extract data on years of work experience, number of keratoconus patients seen per year and access to a topography device. We tested the questionnaire on five subjects to obtain its final form. The relevant ethic committee of Lucerne reviewed the protocol of this study (ReqID-2019-00995) and found that this study did not fall under the Swiss Human Research Act.

### Questionnaire items

The first question assessed participants’ knowledge on the definition of keratoconus, with three possible answers: thinning of the cornea, irregular astigmatism and protrusion of the cornea. In the second question, risk factors for the occurrence of keratoconus, such as positive family history, allergies and younger age were asked for. The third question concerned triggers for the onset of keratoconus, with possible answers comprising eye rubbing, puberty and pregnancy. In the fourth question, participants were requested to name symptoms of keratoconus; possible answers were deterioration of vision, double vision and light sensitivity. The fifth question asked about the consequences of untreated keratoconus, which are visual deterioration, inability to wear glasses, inability to wear contact lenses, restriction of current occupation and need for corneal transplantation. The final question asked for treatment options for keratoconus, such as glasses, rigid contact lenses, corneal cross-linking and corneal transplantation.

### Interviewers

 Two interviewers received an oral and written instruction on how to conduct the interview, and were trained with four test interviewees. Both interviewers were German native speakers and had a professional background as specialized study nurses working in an ophthalmology department. All four test interviewees were board-certified, German-speaking ophthalmologists working in the same ophthalmology department but not members of the corneal unit. The interviewers were specifically instructed not to prompt the interviewees on how many answers to give per question.

### Statistical analysis

Two corneal specialists independently assessed and classified the replies using an assessment sheet with pre-specified correct replies. Statements made during the interview that pointed at a correct reply without perfectly fitting the pre-specified replies were recorded and discussed between the two assessors. Disagreements were resolved by consensus.

The total number of correct answers was 21. The MKK of a person equaled the total number of correct replies multiplied by one hundred divided by 21. In addition, we examined the influence of age (interval scaled), gender (female; male), years of work experience, number of keratoconus patients seen per year and access to a topography device as independent variables and the cumulative proportion of correct replies as the dependent variable using a linear multivariable regression model. We performed the analysis using the Stata 16.1 statistical software package (StataCorp, 4905 Lakeway Drive, College Station, Texas 77,845 USA).

## Results

### Reporting of participant characteristics

In January 2020 we informed 191 ophthalmologists by postal letter about the survey and contacted them 1 week later by telephone. 117 ophthalmologists initially agreed to participate and 100 ophthalmologists (mean age 51.9 years (SD 9.5), 60 % male) were ultimately available for the interview. None of them had to be excluded due to poor German-speaking skills. Two corneal specialists independently assessed and classified the replies: in two cases, consensus was not reached (0.05 %) and the reply was rated as “correct” (i.e., in favor of the ophthalmologist).

The 100 ophthalmologists included in the survey correspond to 17.6 % of ophthalmologists practicing in private practice in the German-speaking part of Switzerland. On average, participants had 16.1 years (SD 9.67) of working experience. The estimated median number of keratoconus patients seen per year was eight (IQR 4.5 to 20), and 60 % of respondents reported having access to a state-of-the-art topography device to detect keratoconus. Fourteen participants (14 %) had experience in surgical interventions such as corneal crosslinking or corneal graft surgery. Demographics of participants are summarized in Table 2.

**Table 2:** Participant demographics

Participants (n = 100)		%/SD/IQR
Mean age (years)	51.9	SD 9.5
Male gender	60	60 %
Mean working experience (years)	16.1	SD 9.67
Median number of keratoconus patients per year	8	IQR 4.5 to 20
Access to topography device	60	50 %
Experience in surgical interventions	14	14 %

### MKK - performance

The mean MKK was 52.0 %, and the range was 28.6–81.0 %. The multivariable analysis assessing the association between participants’ characteristics and MKK found no significant parameter (Table 3). Percentage of correct answers are outlined in Table 1.

**Table 3:** Multivariate analysis assessing the association between participants’ characteristics and MKK

MKK	Coefficent	Standard Error	*p*-value	95 % Confidence Interval
Age	-5.82	3.83	0.13	-13.4 to 1.8
Sex (female)	-1.06	2.83	0.71	-6.7 to 4.6
Access to topography	-3.25	2.92	0.27	-9.1 to 2.6
Average keratoconus patients seen per year	0.08	0.06	0.16	-0.1 to 0.2
Years in private practice	0.34	0.35	0.33	-0.3 to 1.0

### MKK - definitions, risk factors and triggers

Whereas 42 (42 %) of the ophthalmologists recalled that irregular astigmatism and protrusion are two diagnostic indicators for keratoconus, only 24 participants (24 %) recalled all three relevant parameters (including corneal thinning). Of the three most important risk factors for the development of keratoconus, 79 subjects (79 %) correctly stated “positive family history”, while allergies (48 %) and younger age (29 %) were less often recalled. Only 9 % of participants correctly recalled all risk factors. Regarding triggers, eye rubbing was named most often (69 %); puberty (16 %) and pregnancy (4 %) were rarely mentioned.

### MKK - symptoms, consequences of untreated keratoconus

The majority of participants stated that deterioration of vision was an important symptom of keratoconus (97 %), while other important symptoms, including double vision (31 %) and light sensitivity (13 %) were less frequently specified. Regarding consequences of untreated keratoconus, progression of visual deterioration (86 %) was most commonly named. Other consequences, including the requirement of a corneal transplant (36 %), the inability to fit glasses (65 %) or even contact lenses (38 %) were mentioned less often.

Eight respondents acknowledged that keratoconus progression could impede patients from continuing working in their current profession. Five participants correctly answered all questions concerning symptoms, and three participants correctly answered all questions concerning consequences of untreated keratoconus.

### MKK- treatment options

All participants stated that corneal cross-linking was one of the treatment modalities, followed by rigid contact lenses (81 %) and corneal transplant (72 %). Glasses, as the fourth option, was seldom reported (33 %). Twenty participants (20 %) answered all questions regarding treatment options correctly.

## Discussion

### Main findings

In this interview survey we found a substantial mismatch between corneal specialists’ expectations and general ophthalmologists’ knowledge about the typical signs, risk factors and treatment options for keratoconus. Overall, participants recalled only approximately half of the MKK. Contrary to our expectation, a higher number of keratoconus patients seen per year or access to topography devices did not increase MKK.

### Results in light of existing literature

Up to now, little was known about differences in general ophthalmologists’ and corneal specialists’ knowledge regarding chronic eye disease such as keratoconus. Abu-Amara et al. reported a substantial mismatch of expected primary care physicians’ knowledge for the screening and treatment of sight-threatening diabetic retinopathy [14, 15]. In another study, only 52 % of primary care physicians indicated having adequate knowledge to advise their patients on vision health [16]. There is substantial evidence in the medical literature for such differences between generalists and specialists in terms of knowledge, patterns of care and clinical outcomes for a broad range of diseases [17]. Specialists were shown to be more knowledgeable about the management of selected general medicine conditions [15, 18]. Several reasons account for their higher level of knowledge: specialists benefit from treating a narrower range of clinical problems [15], can devote more time to continuing education relevant to such conditions [19] and may have better access to the most recent information than generalists [20]. Given that most ophthalmologists in this cohort see on average one keratoconus patient out of an estimated 700 patients per month in their daily routine, the condition is probably diagnosed too rarely to justify keeping up with the latest developments in the field. Interestingly, some of the replies given by the ophthalmologists were not considered to be MKK, yet were nevertheless correct. Among them, presence of Down Syndrome (31 %) and connective tissue disorders (9 %) were commonly stated risk factors. However, in the group of ophthalmologists naming Down Syndrome as a risk factor, none of the participants named all other risk factors correctly.

### Strengths and limitations

To the best of our knowledge, this is the first survey investigating knowledge about keratoconus in general ophthalmologists.

What are the limitations of this study? We assessed a convenience sample of limited size representing 10 % of all ophthalmologists working in hospitals and private practices in Switzerland. We enrolled only ophthalmologists in private practice willing to participate in the study, which might have introduced selection bias, although we believe that our respondents were in fact more likely to score higher than average, leading to an overestimation of knowledge. We cannot rule out that some particularities in the Swiss continuous education system may impede broad generalizations of our findings to other countries. A further limitation was that we used a non-validated questionnaire. Because no standard and validated questionnaire was available, we designed one according to published recommendations [21]. The questionnaire only fulfilled the element of face validity, which is an important but not sufficient element of questionnaire development. However, the questionnaire was sufficient to point out the substantial mismatch of expected and actual knowledge. Finally, this was a recall test, which is usually more challenging than a recognition test such as multiple-choice exams. Arguably, conducting the interview on the phone created another potential stressor for participants. On the other hand, in our opinion a recall test is more likely to represent the doctors’ behavior in their daily practice than a recognition test. Shared decision making requires knowledge about the disease, justifying our approach [22]. Furthermore, independent research has clearly shown that medical knowledge assessed by a recognition test was also limited in medical students and senior medical educators [23].

### Implications for research and practice

Further research should aim at defining ideal pathways for patient care between general ophthalmologists and corneal specialists in the management of keratoconus. The potential lack of keratoconus knowledge probably reduces awareness of the disease, which impedes patients from being diagnosed at an early stage of the disease and leads to a potentially worse outcome in the long-term. On the other hand, there are still debates among keratoconus specialists about the use of updated classification or surgical management [1]. Therefore, if specialists do not reach a consensus on various aspects of the condition, it is difficult to expect general ophthalmologists to improve their keratoconus management. Nevertheless, there is a need of better general ophthalmologist training on keratoconus to achieve a broader awareness of this condition. Substantial improvement is also needed in interdisciplinary patient care, such as between contact lens specialists, general ophthalmologists and corneal specialists. So far, no established collaborations, networks or common advanced training platforms exist in Switzerland.

## Conclusions

We found a substantial mismatch between corneal specialists’ expectations and general ophthalmologists’ knowledge about keratoconus. Our particular sample had half of the MKK that corneal specialists would expect patients to know, independent of the number of keratoconus patients seen and of access to a state-of-the art topography device. Almost one half of ophthalmologists recalled only one diagnostic criterion of keratoconus, which may be a reason for the low reported keratoconus prevalence in Switzerland compared with in other countries. Under-detection of early keratoconus may lead to delayed intervention and a substantial disease burden of those patients.

## Data Availability

The datasets used and/or analyzed during the current study are available from the corresponding author on reasonable request.

## Abbreviation

MKK: Minimal keratoconus knowledge.
